# Safety of nurse-directed triage intranasal fentanyl protocol for acute pain management in a European pediatric emergency department: A retrospective observational analysis

**DOI:** 10.3389/fped.2023.1070685

**Published:** 2023-02-13

**Authors:** F. Romano, M. Wendelspiess, R. Mansour, O. Abplanalp-Marti, C. Starvaggi, F. Holzner, I. Steiner, K. Keitel

**Affiliations:** ^1^ Department of Paediatrics, Division of Paediatric Emergency Medicine, Inselspital, Bern University Hospital, University of Bern, Switzerland; ^2^ Faculty of Medicine and Medical Sciences, University of Balamand, Beirut, Lebanon

**Keywords:** pediatric emergency medicine, analgesia, pain management, nurse-directed triage protocol, intranasal fentanyl, safety

## Abstract

**Background:**

Nurse-directed pain protocols for intranasal fentanyl administration are not widely implemented in European (EU) pediatric emergency departments (PED). Barriers include perceived safety concerns for intranasal (IN) fentanyl. The aim of this study is to describe our experience with a nurse-directed triage IN fentanyl protocol with a focus on safety in a tertiary EU PED.

**Methods:**

We conducted a retrospective analysis of patient records of children aged 0–16 years who received nurse-directed IN fentanyl between January 2019 and December 2021 at the PED of the University Children's Hospital of Bern, Switzerland. Extracted data points included demographics, presenting complaint, pain score, IN fentanyl dosage, concomitant pain medication use, and adverse events.

**Results:**

A total of 314 patients were identified with ages ranging from 9 months to 15 years. The main indication for nurse-directed fentanyl administration was musculoskeletal pain due to trauma (*n* = 284, 90%). Mild adverse events (vertigo) were reported in two patients (0.6%), without a correlation to concomitant pain medication or protocol violation. The only reported severe adverse event of syncope and hypoxia in a 14-year-old adolescent occurred in a setting where the institutional nurse-directed protocol was violated.

**Conclusion:**

In accordance with previous studies outside of Europe, our data support the case that when appropriately used, nurse-directed IN fentanyl is a safe potent opioid analgesic for pediatric acute pain management. We strongly encourage the introduction of nurse-directed triage fentanyl protocols Europe-wide in order to provide effective and adequate acute pain management in children.

## Introduction

Nurse-directed triage pain protocols (protocols enabling nurses to give analgesics at admission without any medical prescription) have been shown to improve the time to analgesia in emergency medicine (1). Nurse-directed triage pain protocols are essential, especially in pediatric patients, in whom pain is often underrecognized and not adequately addressed before physician contact (2).

However, as recently shown by a survey study conducted by the Research in European Pediatric Emergency Medicine (REPEM) network, in European (EU) pediatric emergency departments (PEDs), triage nurse-directed pain protocols are still not widely implemented (3). Indeed, among those EU PEDs that already have nurse-directed pain protocols in place, only a minority of sites adopt in their protocols intranasal (IN) fentanyl for severe pain management (3), although IN fentanyl represents a potent opioid analgesic providing rapid relief for severe pain without the need for venous access in the PED setting (4, 5). Opioid prescription remains a “physician territory” in Europe, and there are often historical concerns around safety and the apparent lack of specific safety data in the EU PED setting (4, 6).

The standard IN dose of 1.5 µg/kg fentanyl has been shown to provide effective analgesia comparable to the analgesic potency of IV morphine (7, 8). As a selective mu-receptor agonist, fentanyl is safer than other opioids with fewer cardiovascular effects due to a lack of histamine release (9). In standard doses of 1–2 µg/kg, there is no sedative effect and therefore there is a low risk for respiratory depression (5, 8). In addition, when administered IN, fentanyl shows a rapid onset and short duration of action, making it also attractive for prehospital use in children by paramedics (10).

The aim of this retrospective observational study is to describe our experience with a nurse-directed triage IN fentanyl protocol with a focus on safety in a tertiary EU PED.

## Methods

The PED in this study is part of a level 1 pediatric trauma center and university children's hospital with an average of 25,000–30,000 annual presentations of children aged 0–16 years. A nurse-directed triage IN fentanyl protocol has been established since 2013. The protocol allows triage nurses to administer IN fentanyl (standard dose 1.5 µg/kg, max. 100 µg) in patients older than 12 months without prior physician prescription, when severe pain is present. Pain assessment occurs using one of three different pain score scales dependent on age. These are the “Kindliche Unbehagen und Schmerz Skala—KUSS” (<4 years), the Wong–Baker Faces Pain Scale (4–7 years), and the Visual Analog Scale (>8 years). After IN fentanyl administration, oxygen saturation is monitored continuously for 30 min and patients are clinically reassessed by nurses every 15 min (see Supplementary Materials). The nurse-directed IN fentanyl protocol is part of the institutional pain management concept, which is based on the WHO analgesic ladder approach, and allows nurses the administration of non-opioid analgesics for mild and moderate pain or as first-line medication in combination with IN fentanyl.

We retrospectively analyzed the electronic medical records of all children <16 years who received IN fentanyl at triage from January 2019 to December 2021. We extracted data pertaining to demographics, presenting complaint, pain score, concurrent pain medication use, effectively administered IN fentanyl dose, and adverse events. Pain scores were corrected to the Visual Analog Scale, with severe pain defined by a score of ≥6. When adverse events were reported in the electronic medical record, a more detailed chart review was completed in order to identify the relationship with fentanyl administration and to assess the severity of the event (according to fentanyl reference safety information provided by the European Medicine Agency). The vital signs monitoring the record of all included patients were manually screened for any atypical observations that may indicate an adverse event, which was not previously captured. A further search for adverse events was conducted by checking the medication administration record for rescue treatments (e.g., naloxone, volume bolus, etc.).

Continuous variables were described as means ± standard deviations (SD) or median and interquartile range (IQR) and categorical variables as percentages (STATA®.Statistics/Data Analysis, StataCorp LLC).

## Results

A total of 314 patients were identified who received nurse-directed IN fentanyl at triage. During the investigated period, our PED reported 76,043 visits. Table 1 summarizes patient characteristics.

**Table 1:** Collected patient data.

Demographics	
Total number of patients (*N*)	314 (100%)
Sex (*n*)	Male: 199 (63%), female: 115 (37%)
Age (in years)	Mean: 8.4 (SD ± 4, min. 0.8, max. 15.7)
Weight (in kg)	Mean: 32 (SD ± 15.1, min. 9.9, max. 90)
Main complaint at admission	
Burn/scald (*n*)	26 (8.2%)
Isolated injury of the upper extremity (*n*)	211 (67.1%)
Isolated injury of the lower extremity (*n*)	46 (14.6%)
Traumatic injury, other localizations (*n*)	27 (8.6%)
Non-trauma-related pain	3 (1%)
Pain score	
Pain score documented (*n*)	300 (95.5%)
Pain score not documented (*n*)	14 (4.5%)
Pain score (corrected to Visual Analog Scale)	Median: 8 (IQR 6–10)
IN fentanyl dose	
Dosage in µg/kg	Mean: 1.45 (SD ± 0.1, min. 1, max. 2)
Dosage too high, >1.7 µg/kg (*n*)	4 (1%)
Dosage too low, <1.3 µg/kg (*n*)	13 (4%)

In terms of age, the nurse-directed protocol was violated in two cases, in a 9-month-old infant and an 11-month-old one treated for a scald burn. No adverse events were reported in these patients.

Pain in musculoskeletal injury due to trauma was the main indication for nurse-directed IN fentanyl, with isolated injury of the upper extremity being the most reported complaint. Non-trauma-related pain (headache, testicular torsion, torticollis) accounted for only 1% of all treated patients. Interestingly, despite most of the patients receiving IN fentanyl with high pain scores according to the institutional protocol (corrected to Visual Analog Scale scores ≥6), in 28 patients (8.9%), IN fentanyl was administered with lower pain scores. This phenomenon seemed to occur especially when fracture was clinically suspected in anticipation of an x-ray.

One-third of all patients (*n* = 117, 37.3%) had already received analgesics in the 2 h prior admission, of which only three patients (1%) had already received intranasal fentanyl by paramedics. None of these had adverse events. No patient had received other opioids before admission. All patients received IN fentanyl within 10 min from the beginning of triage. Dosing errors with doses exceeding >1.7 µg/kg occurred in four patients (1%), with the maximal administered fentanyl dose being 2 µg/kg. Underdosing (<1.3 µg/kg) in relation to the institutional protocol occurred in 13 patients (4%).

A total of 152 patients (48.4%) received a non-opioid analgesic in addition to IN fentanyl at triage, with non-steroidal anti-inflammatory drugs (NSAIDs) being the most used comedication (*n* = 105, 33.4%) vs. acetaminophen (*n* = 46, 14.6%) and metamizol (*n* = 1, 0.3%).

A second dose of fentanyl was administered in 63/314 (20%) of patients during their PED stay.

Only three adverse events were reported, one of which was categorized as “severe.” The details of the three patients experiencing adverse events are summarized in Table 2.

**Table 2:** Reported adverse events.

** **	**Patient 1**	**Patient 2**	**Patient 3**
Severity	Mild	Mild	Severe
Indication	Forearm fracture	Forearm fracture	Severe hip pain
Age in years	12.5	12.8	14.2
Weight in kg	40	47	90
Chronic condition	None	None	None
Administered IN fentanyl dose in µg/kg	1.5	1.4	1.9
Comedication	None	None	None
Adverse event	Vertigo	Vertigo	Syncope, hypoxia
Necessary measures	None, self-limited	None, self-limited	O_2_-support with rapid recovery

One severe adverse event was reported in a 14-year-old, previously healthy patient. IN fentanyl was used to mobilize the patient with severe hip pain out of the parent's car. The patient received two consecutive doses: 120 µg (1.3 µg/kg) and an additional 50 µg (0.6 µg/kg) within 5 min. After a further 10 min, the patient could be mobilized but experienced a syncope and transient desaturation on pulse oximetry requiring short-term oxygen support (description in the electronic medical record, minimum level of SpO_2_ not specified). The patient showed rapid recovery, and the administration of naloxone or volume bolus was not needed. Globally seen, this severe adverse event occurred in an atypical setting and the IN fentanyl administration breached the conditions of our institutional nurse-directed pain protocol (first dose exceeding the maximum absolute dose of 100 µg, second dose after 5 min and administered *via* medical prescription and not nurse-directed).

## Discussion

We presented our experience of IN fentanyl use at triage through a nurse-directed pain protocol in an EU PED setting. In our retrospective cohort study, we could show that IN doses of 1.3–1.7 µg/kg are not associated with relevant adverse events in accordance with previous evidence outside of EU PEDs (4, 5, 11, 12).

Our findings are supported by two previous studies on nurse-initiated IN fentanyl protocols in American and Australian PEDs (11, 12). In these settings, no safety concerns were reported and IN fentanyl was found to be effective for early pain relief. Numerous studies have demonstrated the safety and efficacy of IN fentanyl in physician-directed protocols, including procedural sedation (8, 9, 13, 14). However, previous studies reported data limited to children >3 years. We included a total of 34 patients aged ≤36 months, two of whom were younger than 12 months (protocol violation). No adverse events were observed in this younger age group. IN fentanyl use in children less than 3 years has also been reported to be safe in prehospital settings (10).

Our protocol uses an IN fentanyl dose of 1.5 µg/kg. A total of 20% of all patients required repeated IN fentanyl doses during the course of their stay at PED, which suggests that this dose is generally efficacious. This is especially true when IN fentanyl is used in combination with over-the-counter analgesics such as NSAIDs and acetaminophen. In our cohort, 36.3% of patients had received non-opioid analgesics prior to arrival at the PED and 48.4% of them received first-line medications in addition to fentanyl at triage. This is in accordance with the WHO analgesic ladder approach and with international pediatric pain management guidelines, which recommend the use of fentanyl as a co-analgesia for moderate and severe pain with first-line medications, in order to sustain pain relief and to achieve an opioid-sparing effect with reduced adverse events for the patient (15).

Dosing errors occurred in 5% of patients, and only 1% were administered a dose that exceeded the protocol-defined dosage. Except for one case of overdosing due to a protocol violation, all dosing errors occurred because of incongruence between the patient's weight that was verbally reported by parents in triage and the actual patient's weight measured later on in the consultation. A reported weight is often used in triage in order to not delay time to drug administration and to avoid further pain during the weighting process. Although weight is often underestimated in children, as reflected by more underdosing errors in our cohort, IN fentanyl seems to have the advantage over other opioid agents with a narrower therapeutic index, in that safety is maintained even with higher-than-target doses and repeat dosing (10, 11).

The unique reported severe adverse event in our cohort occurred in a patient where the protocol was violated with the administration of repeated doses of fentanyl within 5 min and with the first administration exceeding the absolute dose of 100 µg. While adverse events from fentanyl can include hypotonia and respiratory depression, the rapid spontaneous resolution of the symptoms without the need for the administration of a volume bolus or of naloxone in our patient supports the hypothesis that vasovagal syncope occurred possibly because of pain. In previous studies, repeated IN fentanyl doses exceeding the total dose of 2 µg/kg have not been shown to be associated with severe adverse events (11).

In this regard, one limitation of our study is the lack of a systematic assessment of adverse events. This is principally due to the retrospective nature of our study, but also due to a lack of a digitalized safety reporting system specific for medications in our institution. In consideration of the fact that the review process for adverse events consisted in the analysis of the patients' electronic medical records as well as of the monitoring and medication administration records, we expect that no severe adverse events were missed in our cohort. However, mild adverse events may be underrepresented in our study if they had not been properly documented.

Another limitation of our study is selection bias: the main indication for nurse-directed fentanyl administration in our cohort was musculoskeletal pain due to trauma in previously healthy patients. Although IN fentanyl has been shown to be effective and safe in patients with chronic illness in the case of vaso-occlusive pain in sickle-cell disease, further research is needed to expand the use of nurse-directed intranasal fentanyl protocols to pediatric patients with acute severe pain in underlying chronic disease (16).

An interesting finding of our study was that 9% of all patients received IN fentanyl despite reporting low pain scores (Visual Analog Scale <6). A detailed chart review of these patients showed that IN fentanyl was given in anticipation of expected pain during x-ray. Anticipatory pain treatment appears to be an important indication in nurse-directed pain management protocols (1). In comparison with adults, pain in children is often undertreated, especially because of communication challenges and out of fear of adverse effects from analgesic medications (2). The availability of IN fentanyl protocols has been shown to increase the awareness of the need for providing pain relief in children, resulting in a reduction in time to pain treatment (17). Holdgate et al. also demonstrated that it also leads to an increase in the proportion of young children treated for severe pain overall (17).

Although nurse-directed pain protocols in general have the potential for providing more effective pain relief in the PED setting and sensitization of caregivers toward adequate pain management for their children, these protocols are still not widely implemented. In a survey among 171 university hospitals and tertiary care centers of 19 EU countries, nurse-directed pain protocols were available only in 53% of all sites and IN fentanyl only in 11 sites (6%) (2). Important barriers to implementation include the perceived safety of nurse-directed IN fentanyl protocols (18).

In conclusion, on the basis of our data on nurse-directed IN fentanyl protocol in an EU PED setting, combined with previous evidence on this topic outside Europe, as well as the evidence available on the efficacy and safety of IN fentanyl in general, we encourage the implementation of nurse-directed IN fentanyl pain protocols in EU PED to improve pediatric acute pain management.

## Data Availability

The data analyzed in this study are subject to the following licenses/restrictions: Institutional and privacy restrictions. Requests to access these datasets should be directed to Fabrizio Romano, fabrizio.romano@insel.ch.
