# Supplementary material for: Safety of nurse-directed triage intranasal fentanyl protocol for acute pain management in a European pediatric emergency department: A retrospective observational analysis
Source: Front Pediatr. 2023 Feb 13;11:1070685. doi: 10.3389/fped.2023.1070685 (PMC9969078; doi:10.3389/fped.2023.1070685)
Supplement: Supplementary file 1 [file Datasheet1.pdf]

Supplement

Nurse-directed intranasal fentanyl protocol
(translated from institutional original German protocol)

Indication

- Children ≥ 1 year of age, moderate to severe pain, e.g. fractures of the long bones, joint dislocations, burns/scalds

Contraindications

- Children less than 1 year of age, suspicion or confirmation of fracture of the base of the skull, known intolerance or allergy to Fentanyl

Safety/Monitoring

- Double check weight-dependent dose
- Be prepared for O_2-administration and airway support
- Monitoring after single dose:
    - Documentation of baseline values (pulse and saturation)
    - If baseline vital signs are not in the normal range, question the reason and discuss the situation with a supervisor (consultant/senior physician)
    - Until 30 minutes after drug administration: continuous pulse oximetry, clinical assessment every 15 minutes by the nurse.
    - Stop monitoring after 30 minutes if the patient's vital signs are in the normal range and the patient is not complaining about side effects
- Consider naloxone if severe adverse events (discuss with consultant/senior physician)

Fasting time

- Not necessary

Material

- Fentanyl Inj. Solution Amp 50mcg/ml
- 1ml- or 2ml-syringe topped with the MAD Nasal™ IN Mucosal Atomization Device (LMA®)

Dose

- 1.5mcg fentanyl/kg body weight as initial dose (0.03ml/kg of the 50mcg/ml dilution). For weights over 33kg (>1ml) administration divided between both nostrils. For weights over 66kg administration of first absolute dose of 100ug (1ml per nostril), the remaining dose should be administered after 15 minutes.
- If persistent severe pain, after 15 minutes at the earliest, administer a second dose of max. 0.5mcg/kg after consulting the senior physician.

Weight	Dose in mcg	Dose in ml
10 kg	15 mcg	0.3 ml
15 kg	22.5 mcg	0.45 ml
20 kg	30 mcg	0.6 ml
25 kg	37.5 mcg	0.75 ml
30 kg	45 mcg	0.9 ml
35 kg	52.5 mcg	1.05 ml
40 kg	60 mcg	1.2 ml
45 kg	67.5 mcg	1.35 ml
50 kg	75 mcg	1.5 ml
55 kg	82.5 mcg	1.65 ml
60 kg	90 mcg	1.8 ml
65 kg	97.5 mcg	1.95 ml
70 kg	105 mcg	2.1 ml
75 kg	112.5 mcg	2.25 ml
80 kg	120 mcg	2.4 ml

Procedure

- First inspect the nose for blood or secretions. If present, clean or suction if necessary. If persistent, switch to other route of administration (i.v.).
- Draw up sufficient Fentanyl solution in the syringe. Fill the MAD with the fentanyl solution (otherwise the dead space of 0.1ml will not be administered). Then empty with the MAD in place the superfluous fentanyl volume, until the desired dose is in the syringe.
- Stabilize the head with the free hand. Insert the MAD into the nostril and administer fentanyl by orientating the syringe in direction of the patient's ear.

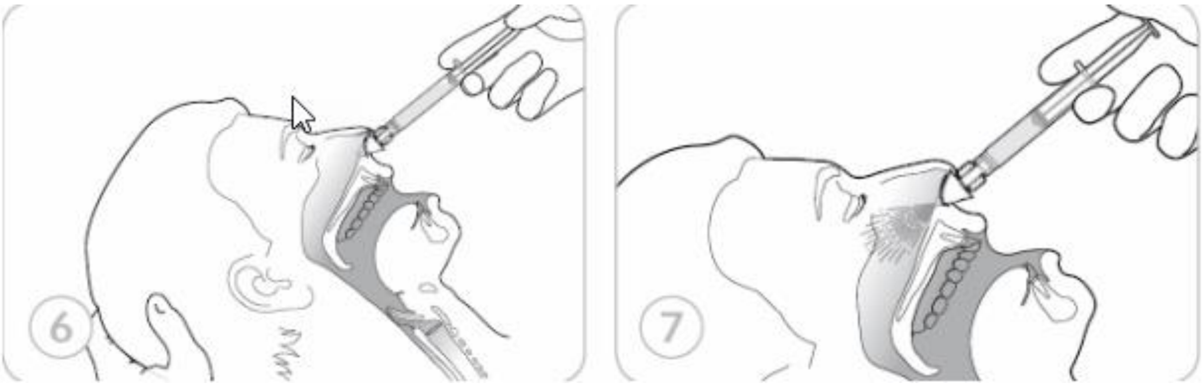

Side effects

- nausea, vomiting
- dizziness, blurred vision
- respiratory depression, apnea
- bradycardia, hypotension (rare)
- chest wall rigidity (described only in i.v. administration)

Documentation of pain intensity:

- Use respective pain scores according to the institutional pain management concept
